# Baicalin Ameliorates H_2_O_2_ Induced Cytotoxicity in HK-2 Cells through the Inhibition of ER Stress and the Activation of Nrf2 Signaling

**DOI:** 10.3390/ijms150712507

**Published:** 2014-07-15

**Authors:** Miao Lin, Long Li, Yi Zhang, Long Zheng, Ming Xu, Ruiming Rong, Tongyu Zhu

**Affiliations:** 1 Department of Urology, Fudan University Zhongshan Hospital, Shanghai 20032, China; E-Mails: linmiao@fudan.edu.cn (M.L.); xu.ming@zs-hospital.sh.cn (M.X.); 2 Shanghai Key Laboratory of Organ Transplantation, Shanghai 20032, China; E-Mails: ljlmjw@163.com (L.L.); yzhang_med@fudan.edu.cn (Y.Z.); longzheng0814@126.com (L.Z.)

**Keywords:** Baicalin, kidney, ischemia-reperfusion injury, tubular epithelial cells, ER stress, Nrf2

## Abstract

Renal ischemia-reperfusion injury plays a key role in renal transplantation and greatly affects the outcome of allograft. Our previous study proved that Baicalin, a flavonoid glycoside isolated from *Scutellaria baicalensis*, protects kidney from ischemia-reperfusion injury. This study aimed to study the underlying mechanism *in vitro*. Human renal proximal tubular epithelial cell line HK-2 cells were stimulated by H_2_O_2_ with and without Baicalin pretreatment. The cell viability, apoptosis and oxidative stress level were measured. The expression of endoplasmic reticulum (ER) stress hallmarks, such as binding immunoglobulin protein (BiP) and C/EBP homologous protein (CHOP), were analyzed by western blot and real-time PCR. NF-E2-related factor 2 (Nrf2) expression was also measured. In the H_2_O_2_ group, cell viability decreased and cell apoptosis increased. Reactive Oxygen Species (ROS) and Glutathione/Oxidized Glutathione (GSH/GSSG) analysis revealed increased oxidative stress. ER stress and Nrf2 signaling also increased. Baicalin pretreatment ameliorated H_2_O_2_-induced cytotoxicity, reduced oxidative stress and ER stress and further activated the anti-oxidative Nrf2 signaling pathway. The inducer of ER stress and the inhibitor of Nrf2 abrogated the protective effects, while the inhibitor of ER stress and the inducer of Nrf2 did not improve the outcome. This study revealed that Baicalin pretreatment serves a protective role against H_2_O_2_-induced cytotoxicity in HK-2 cells, where the inhibition of ER stress and the activation of downstream Nrf2 signaling are involved.

## 1. Introduction

Renal ischemia-reperfusion injury (IRI) is an inevitable acute injury during renal transplantation, which greatly affects the outcome of allograft [1,2]. Renal tubular epithelial cells (TECs) suffer from cell injury and undergo apoptosis in renal IRI, contributing to the loss of graft function. The pathophysiological change and underlying mechanism of TECs apoptosis are quite complex [3], involving the caspase cascade, mitochondria dysfunction, immune injury and endoplasmic reticulum (ER) stress [4]. The prevention of TECs apoptosis is considered to be an effective method to alleviate renal IRI.

It is widely accepted that ER stress plays a key role during the progress of kidney injury and TEC apoptosis [5]. The accumulation of unfolded/misfolded proteins, calcium depletion and energy deprivation during IRI break the equilibrium in ER and constitute a form of stress in the cell—the ER stress [6]. A serial of conservative and complementary adaptive signaling pathways were activated to cope with the perturbations, which are generally called the unfolded protein response (UPR). UPR is classically related to controlled translation, decreased protein load and upregulated chaperones, including binding immunoglobulin protein (BiP). The homeostasis in the ER is maintained through the adaptive response of UPR [7]. However, IRI-induced ER stress is too severe to overcome. UPR gradually develops into the apoptosis phase in renal IRI. C/EBP homologous protein (CHOP) accumulation, IRE1 phosphorylation and JNK activation initiate the apoptosis phase and finally lead to cell apoptosis in renal IRI [8]. BiP and CHOP are generally considered as hallmarks of the ER stress level.

NF-E2-related factor 2 (Nrf2) activation plays a key role in UPR. Nrf2 has been proven to be a master transcriptional regulator of the expression of genes coding detoxification enzymes, antioxidant proteins and other stress response mediators [9]. Liu *et al.* reported that Nrf2-deficiency enhances the susceptibility of mice to ischemic kidney injury and identified Nrf2 as a protective transcription factor in 2009 [10]. The adaptive response of UPR recruits diverse molecules and pathways to compensate the stress condition. There is a crosstalk between ER stress and Nrf2, and Nrf2 can be activated by ER stress [11]. The IRI-ER stress-Nrf2 axis exerts important protective effects to kidney.

Baicalin is a flavonoid glycoside extracted from a kind of traditional Chinese drug, *Scutellaria baicalensis*, and its chemical structure has been clarified [12]. Baicalin is proven to possess anti-bacterial, anti-inflammatory and anti-apoptosis properties and is widely used in the treatment of infectious and inflammatory diseases [13,14]. We previously reported that Baicalin pretreatment protects rat kidney from IRI and inhibits inflammation and TEC apoptosis [15]. However, the underlying mechanisms are still unclear. Given the important role of ER stress and Nrf2 in renal IRI, we hypothesized that the protection of Baicalin against renal IRI is mediated by decreased ER stress and activated Nrf2 signaling. To test this hypothesis, HK-2 cells were cultured under the stimulation of H_2_O_2_ with and without Baicalin pretreatment. The ER stress level, Nrf2 activation and cell apoptosis were measured. Our data showed that Baicalin pretreatment suppresses ER stress and activates Nrf2 signaling *in vitro*.

## 2. Results

### 2.1. Baicalin Pretreatment Increased Cell Viability after H_2_O_2_ Stimulation

**Figure 1:**
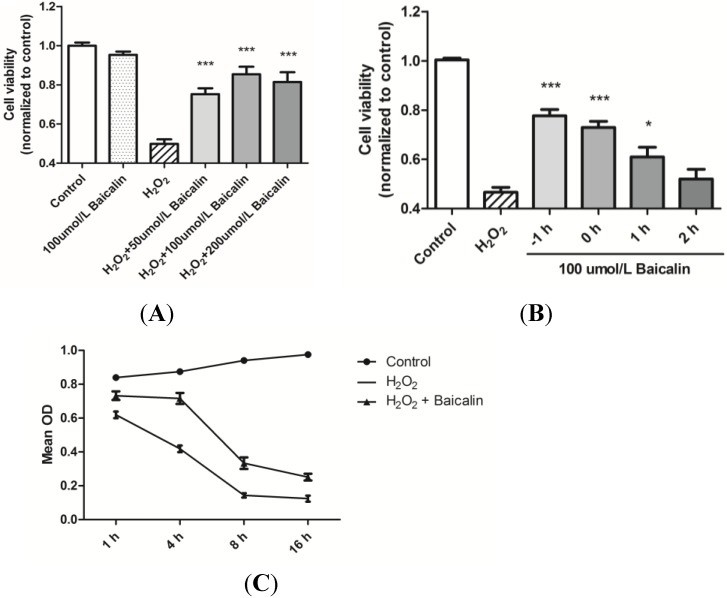
Effects of Baicalin on the cell viability in HK-2 cells by the CCK-8 assay. HK-2 cells were treated with H_2_O_2_ and Baicalin together or separately. The effects of Baicalin treatment were evaluated under different concentrations (**A**) and start times of incubation (**B**) The change of cell viability was also analyzed along with time (**C**). The 1 h pretreatment of 100 μmol/L of Baicalin exerted the best protective effects against H_2_O_2_-induced cell injury (**A**, **B**); Additionally, the largest difference in cell viability between the H_2_O_2_ + Baicalin group and the H_2_O_2_ group was observed after 4 h of stimulation (**C**). * *p* < 0.05 and *** *p* < 0.001, *vs.* the group with the H_2_O_2_ treatment, *n* = 6.

HK-2 cells treated with H_2_O_2_ were assigned to different groups according to different pretreatment concentrations of Baicalin (Figure 1A). Results were compared between the control group and the Baicalin groups. Experiments showed that Baicalin treatment alone had no effect on the cell viability, while Baicalin pretreatment exerted protective effects on cell viability after H_2_O_2_ stimulation. Additionally, the best protective effect was observed at the dosage of the 100 μmol/L Baicalin pretreatment. The protective effects of Baicalin were further evaluated according to the start time of Baicalin incubation (Figure 1B). Results showed that earlier treatment of Baicalin was positively correlated with higher cell viability. In order to observe the change of cell viability along with H_2_O_2_ stimulation time, we compared the Optical density (OD) value by CCK-8 assay at different time points after H_2_O_2_ stimulation (Figure 1C). The largest difference of cell viability between the H_2_O_2_ + Baicalin group and the H_2_O_2_ group was observed after 4 h of stimulation, which suggested that 4 h of H_2_O_2_ stimulation is most appropriate for our study. Thus, we decided to choose the 100 μmol/L Baicalin pretreatment and 4 h of H_2_O_2_ stimulation in the following studies.

### 2.2. Baicalin Pretreatment Decreased Oxidative Stress after H_2_O_2_ Stimulation

**Figure 2:**
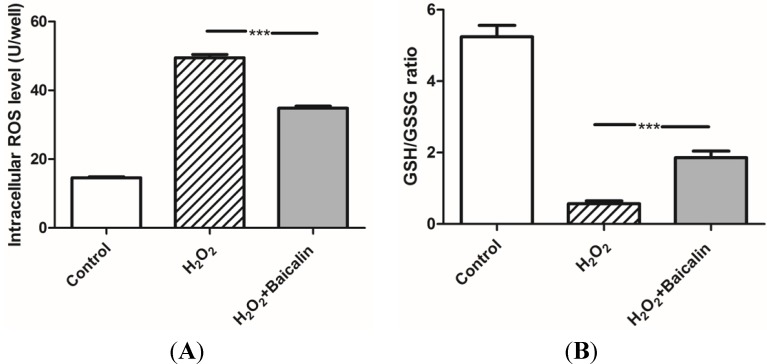
The Reactive Oxygen Species (ROS) level and Glutathione/Oxidized Glutathione (GSH/GSSG) ratio in the cultured HK-2 cells (**A**). The ROS level was measured in cultured HK-2 cells. The results showed that H_2_O_2_ treatment greatly increased the cellular ROS level, while additional Baicalin pretreatment inhibited the increase of the ROS level; and (**B**) The GSH/GSSG ratio was analyzed in cultured HK-2 cells. The GSH/GSSG ratio was down-regulated by H_2_O_2_ treatment, while additional Baicalin pretreatment up-regulated the GSH/GSSG ratio. *** *p* < 0.001, *vs.* the group with H_2_O_2_ treatment, *n* = 6.

The ROS level and Glutathione/Oxidized Glutathione (GSH/GSSG) ratio reflect the oxidative stress in cells. H_2_O_2_ treatment greatly induces oxidative stress and is widely applied to simulate IRI *in vitro*. Our experiments showed that HK-2 cells pretreated with 100 μmol/L of Baicalin had a better ability to resist H_2_O_2_ injury and low intracellular oxidative stress than cells in the H_2_O_2_ group (Figure 2).

### 2.3. Baicalin Pretreatment Inhibited Caspase-3 Activation and Cell Apoptosis after H_2_O_2_ Stimulation

**Figure 3:**
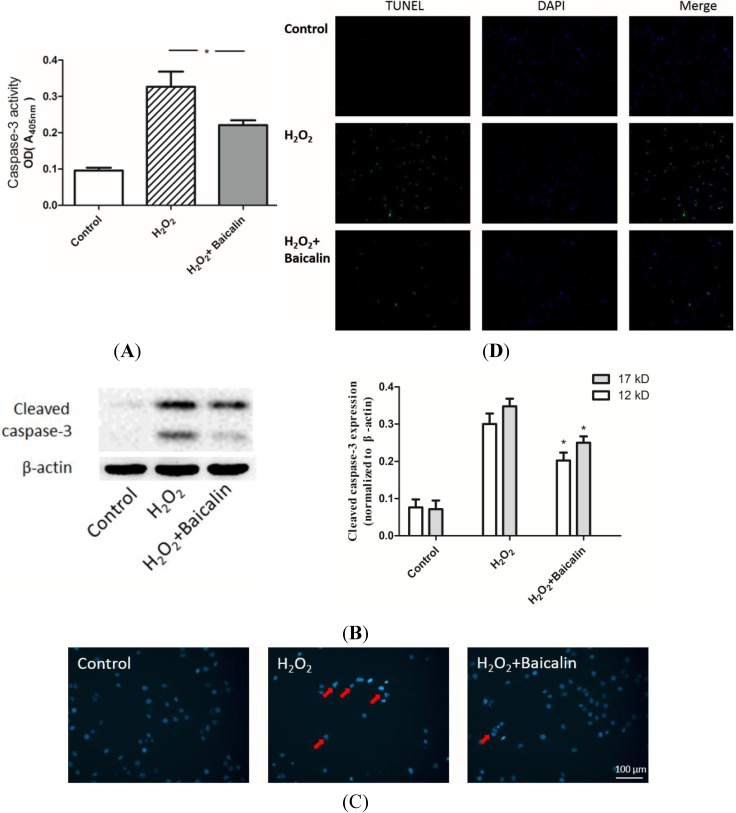
Effects of Baicalin on H_2_O_2_-induced apoptosis in HK-2 cells. The activity of caspase-3 (**A**) and the expression of cleaved caspase-3 (**B**) were evaluated in cultured HK-2 cells. H_2_O_2_ treatment up-regulated both the activity of caspase-3 and the expression of cleaved caspase-3. Baicalin pretreatment suppressed H_2_O_2_ and induced up-regulation of caspase-3 activity and cleavage; Cultured HK-2 cells were stained with Hoechst (**C**) Apoptotic cells showed nucleic fragmentation with dense chromatin (red arrow); The transferase-mediated dUTP-biotin nick end labeling (TUNEL) assay revealed more positive staining cells (with green fluorescence) in the H_2_O_2_ group than in the H_2_O_2_ + Baicalin group (**D**). H_2_O_2_ treatment increased cell apoptosis, while Baicalin pretreatment suppressed cell apoptosis. * *p* < 0.05, *vs.* the group with H_2_O_2_ treatment, *n* = 6.

The caspase cascade induces cell apoptosis after various injuries. Caspase-3 is a downstream effector in this cascade, which directly mediates apoptosis when activated by various upstream signals. Thus, caspase-3 is regarded as a pivotal indicator. We found that Baicalin pretreatment significantly inhibited the activity of caspase-3 in HK-2 cells after 4 h of H_2_O_2_ stimulation (Figure 3A). Cells were then cultured up to 24 h, and the expression of cleaved caspase-3 was analyzed from extracted protein (Figure 3B). Hoechst staining showed that cell nucleus in the H_2_O_2_ group exhibits a brighter color and more smashed staining than the cell nucleus in the H_2_O_2_ + Baicalin group, which suggests less apoptosis with Baicalin pretreatment (Figure 3C). The results of the transferase-mediated dUTP-biotin nick end labeling (TUNEL) assay were in accordance with the results of Hoechst staining (Figure 3D). Less positive cells were observed in the Baicalin pretreatment group.

### 2.4. Baicalin Pretreatment Reduced Endoplasmic Reticulum (ER) Stress Induced by H_2_O_2_ Stimulation

**Figure 4:**
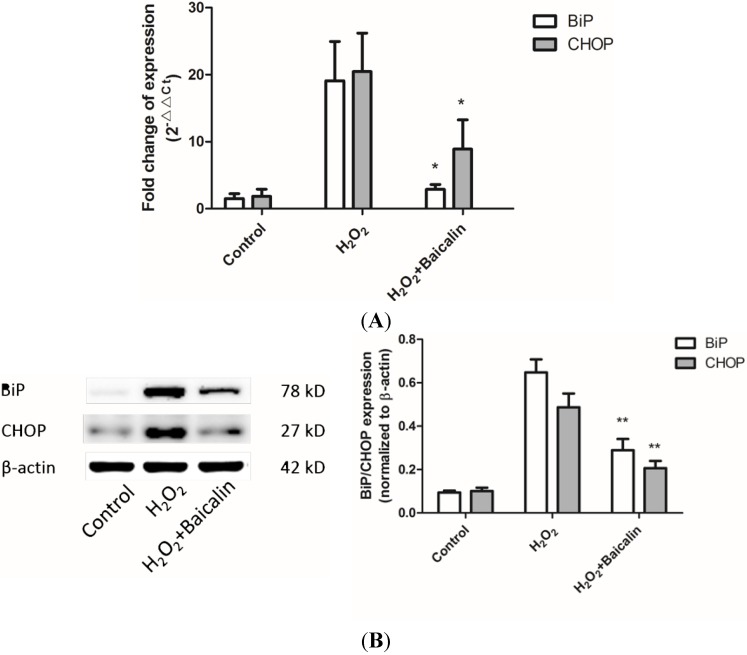
Effects of Baicalin on ER stress hallmarks in cultured HK-2 cells after H_2_O_2_ treatment. Real-time PCR (**A**) and Western blot (**B**) analysis showed increased expression of ER stress hallmarks, binding immunoglobulin protein (BiP) and C/EBP homologous protein (CHOP), after H_2_O_2_ treatment. Baicalin pretreatment inhibited H_2_O_2_ induced upregulation of the expression of ER stress hallmarks. * *p* < 0.05 and ** *p* < 0.01, *vs.* the group with H_2_O_2_ treatment, *n* = 6.

ER stress can be generally assessed by the BiP and CHOP level. Western blot and real-time PCR analysis revealed that Baicalin pretreatment could reduce the mRNA and protein level of BiP and CHOP, indicating reduced ER stress in HK-2 cells under H_2_O_2_ stimulation (Figure 4).

### 2.5. Baicalin Pretreatment Reduced ER Stress Induced by Tunicamycin Stimulation

**Figure 5:**
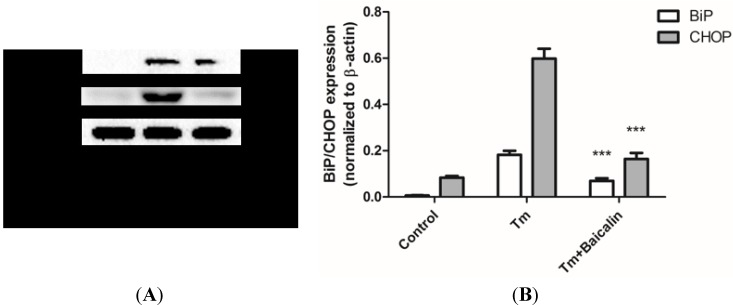
The effects of Baicalin on ER stress hallmarks in cultured HK-2 cells after tunicamycin treatment. HK-2 cells were treated with 2.5 μg/mL tunicamycin for 24 h. Tunicamycin treatment increased expression of BiP and CHOP (**A**); indicating the up-regulation of ER stress (**B**). Baicalin pretreatment significantly decreased tunicamycin-induced up-regulation of the expression of BiP and CHOP (**A**, **B**). *** *p* < 0.001, *vs*. the group with H_2_O_2_ treatment, *n* = 6.

We used the tunicamycin (Tm)-induced ER stress model to study the direct inhibition of Baicalin pretreatment against ER stress. The BiP and CHOP levels were down-regulated by Baicalin pretreatment. Results confirmed that Baicalin pretreatment reduces tunicamycin-induced ER stress (Figure 5).

### 2.6. Baicalin Pretreatment Promoted the Expression of Nrf2 Induced by H_2_O_2_ Stimulation

**Figure 6:**
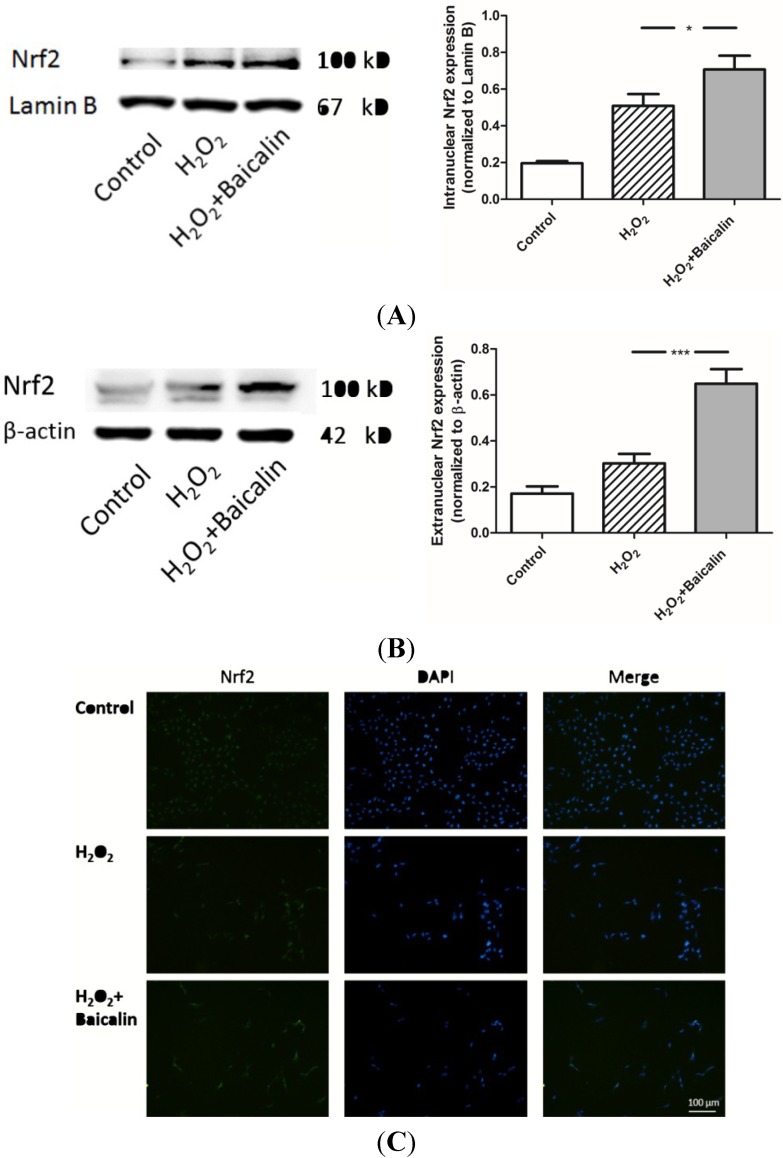
Intranuclear and extranuclear expression of Nrf2 in cultured HK-2 cells. Western blot analysis showed both intranuclear (**A**) and extranuclear (**B**) Nrf2s were increased by H_2_O_2_ treatment; In addition, Baicalin pretreatment further promoted the expression of Nrf2. Immunocytochemistry (**C**) showed that a brighter color of Nrf2 is observed in the H_2_O_2_ + Baicalin group than the color in the H_2_O_2_ group. * *p* < 0.05 and *** *p* < 0.001, *vs.* the group with H_2_O_2_ treatment, *n* = 6.

Nrf2 is accepted as an important effector of the adaptive response of UPR and a key antioxidant transcription factor in eukaryotes. We extracted cytoplasmic and intranuclear proteins from HK-2 cells and measured the protein level in different groups. Results showed that H_2_O_2_ stimulation up-regulated both the cytoplasmic and intranuclear expression of Nrf2, which were possibly related to the cell adaptive response. Baicalin pretreatment further promoted the expression of antioxidant transcription factor Nrf2, suggesting that the adaptive response of UPR was further activated by Baicalin treatment (Figure 6).

### 2.7. The Influences of ER Stress and Nrf2 Regulation on Baicalin Mediated Protection against H_2_O_2_-Induced Injury

In order to clarify the relationship between the protective role of Baicalin and the change of ER stress and Nrf2, we used inhibitors and inducers of ER stress and Nrf2 expression to observe their influences on the protection mediated by Baicalin pretreatment. Tauroursodeoxycholic acid dihydrate (TUDCA) is generally accepted as an inhibitor of ER stress [16], while Tm is a pharmacological inducer of ER stress. Brusatol is the inhibitor of Nrf2 [17] and *tert*-butylhydroquinone (*t*-BHQ) is the inducer of Nrf2 [18]. These chemical reagents were applied in combination with Baicalin. Cell viability, ER stress and intranuclear Nrf2 were then analyzed. Results showed that Baicalin-mediated protection was abrogated by Tm and brusatol, which suggested that the inhibition of ER stress and the activation of Nrf2 were essential for Baicalin-mediated protection. However, Baicalin pretreatment in combination with TUDCA and *t*-BHQ did not have extra effects on cell viability compared with Baicalin pretreatment alone. This indicated that Baicalin shares some common targets with TUDCA and *t*-BHQ: ER stress and Nrf2 expression. These results confirmed that Baicalin protects HK-2 cells from H_2_O_2_-induced cytotoxicity through the inhibition of ER stress and the activation of Nrf2.

## 3. Discussion

As a flavonoid glycoside derived from a kind of traditional Chinese drug, *Scutellaria baicalensis*, Baicalin has been proven to protect rat kidney from IRI through the inhibition of apoptosis and inflammation according to our previous research [15]. Results of the research showed that Baicalin treatment could significantly decrease the apoptosis of TECs and down-regulate tissue oxidative stress after IRI. In order to further investigate the underlying mechanism of Baicalin protection, we chose H_2_O_2_-stimulated HK-2 cells to simulate renal IRI *in vitro* and studied the influence of Baicalin on ER stress. We found that Baicalin ameliorates H_2_O_2_-induced cytotoxicity in HK-2 cells through the inhibition of ER stress and the activation of Nrf2 signaling.

Tubular epithelial cells are the main targets of IRI. Their apoptosis results in the loss of kidney function and delayed graft function during renal transplantation [19]. ER stress has been considered as one main cause that induces the apoptosis of TECs [20,21]. ER is an important organelle, which is responsible for the synthesis, modification and secretion of proteins. ER stress can be induced by many factors, including calcium imbalance, oxidative stress and energy deprivation. Moderate ER stress can trigger adaptive responses, which reduce the protein burden and increase the ER capacity [7]. However, IRI induced ER stress is too intensive and finally leads to cell apoptosis, where CHOP has been proven to play a key role [5,6,21]. This experiment was designed to validate the hypothesis that the protection of Baicalin against renal IRI is mediated by decreased ER stress and activated Nrf2.

We first verified the protective effects of Baicalin in HK-2 cells. The cell viability of HK-2 cells stimulated by H_2_O_2_ was detected by the CCK-8 assay (Figure 1). Additionally, a significant increase in cell viability was observed after Baicalin treatment. We compared the results under different concentrations of Baicalin pretreatment to find an appropriate concentration with the best protective effects. It was proven that 100 μmol/L of Baicalin was the most effective among all of these groups and was thus adopted in the following experiments. We also compared cell viability under different start times of Baicalin incubation after H_2_O_2_ stimulation. Results showed that 1 h of pretreatment of Baicalin displayed the best protective effect. Then, we detected the change of cell viability along with the time of H_2_O_2_ stimulation duration and found that Baicalin had the best protective effects after 4 h of H_2_O_2_ stimulation. According to these results, 1 h of pretreatment of 100 μmol/L Baicalin was adopted in the HK-2 cell model with 4 h of H_2_O_2_ stimulation.

The results of Hoechst staining and the TUNEL assay showed that the apoptosis of TECs was lower in the H_2_O_2_ + Baicalin group than in the H_2_O_2_ group. Decreased apoptosis is associated with the deactivation of a caspase cascade. Caspase-3 is a downstream effector in this cascade, directly mediating apoptosis when activated by various upstream signals [22,23]. We first analyzed the activity of caspase-3, which suggested that Baicalin pretreatment down-regulated the activity of caspase-3 under H_2_O_2_ stimulation. The cleavage of caspase-3 is another typical marker of cell apoptosis. Additionally, it was also analyzed in our experiment to confirm that Baicalin inhibited cell apoptosis (Figure 3). In the treatment group, cells expressed a lower level of cleaved caspase-3 than the level of cleaved caspase-3 in the H_2_O_2_ group. Oxidative stress, which generates the initial injury to HK-2 cells in our experiment, was also evaluated. The ROS level and GSH/GSSG ratio showed that Baicalin pretreatment decreased cellular oxidative stress (Figure 2). These results showed that Baicalin could decrease oxidative stress and reduce cell apoptosis in HK-2. Therefore, it is conceivable that Baicalin protects HK-2 cells from H_2_O_2_-induced cytotoxicity.

In order to study the underlying mechanism, ER stress and one of its main downstream factor, Nrf2, were discussed here. As an important chaperone in ER lumen, BiP interacts with polypeptide folding and controls the structural maturation of nascent glycoproteins [24]. Besides, BiP is also a stress protein, whose expression level is closely related to the intensity of ER stress [25]. CHOP is another hallmark of ER stress intensity [25]. Researchers have proven CHOP as an important factor during ER stress-induced apoptosis. The deletion of CHOP leads to reduced apoptosis, and over-expression of CHOP increases cell apoptosis [26,27,28]. We demonstrated that Baicalin pretreatment could effectively reduce the expression of BiP and CHOP (Figure 4). This result suggested a decrease of ER stress by Baicalin pretreatment and a possible mechanism of reduced HK-2 cell apoptosis: the down-regulation of CHOP. When an intensive ER stress was ameliorated, UPR could switch from the apoptosis phase to adaptive responses [29]. Nrf2 is a transcription factor mostly known by its induction through an antioxidant response [30]. It can also be activated by adaptive responses of UPR machinery. In the context of ER stress, PERK has been suggested as a Nrf2 activator in many articles and the IRE1α-JNK-Nrf2 axis also seems to have an influence on Nrf2 activation [11]. Considering the important effects of Nrf2 in renal IRI [10], we measured Nrf2 to assess the adaptive responses of URP. Our research showed that the intracellular and extracellular levels of Nrf2 were both up-regulated (Figure 6), indicated that Nrf2 was greatly activated by Baicalin treatment. Our results proved that Baicalin promotes the adaptive response of UPR.

**Figure 7:**
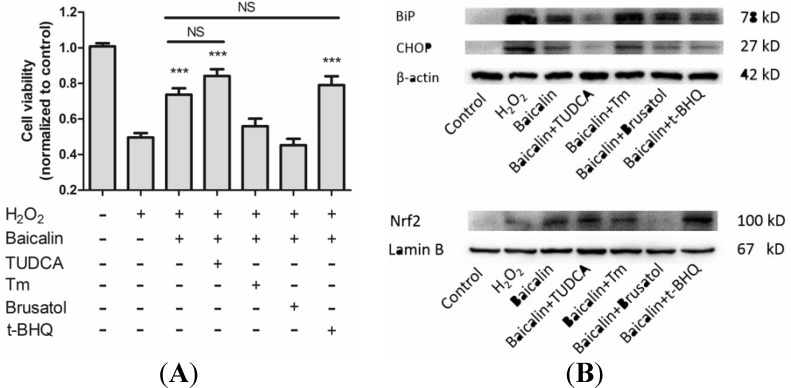
ER stress and Nrf2 regulation in Baicalin-mediated protection. We used ER stress inhibitor tauroursodeoxycholic acid dihydrate (TUDCA) (100 μM), ER stress inducer Tm (2.5 μg/mL), Nrf2 inhibitor brusatol (80 μM) and Nrf2 inducer *tert*-butylhydroquinone (*t*-BHQ) (40 μM) in combination with Baicalin pretreatment to study the role of ER stress and Nrf2 in Baicalin-mediated protection. Western blot analysis proved their effects on ER stress and Nrf2 expression (**B**); We then compared the cell viability between different groups (**A**). There was no significant difference between Baicalin pretreatment with TUDCA or the *t*-BHQ group and the Baicalin pretreatment alone group. These three groups both have higher cell viability than the cell viability in the H_2_O_2_ group. However, Baicalin pretreatment with Tm or brusatol did not exert protective effects against H_2_O_2_-induced injury. These two groups did not have higher cell viability than the cell viability in the H_2_O_2_ group. *** *p* < 0.001 *vs*. the group with H_2_O_2_ treatment, *n* = 6.

However, it was still not clear what was the role of ER stress and Nrf2 during the Baicalin-mediated protection. Whether the Baicalin-mediated decrease of ER stress is direct or indirect is not certain. It is possible that Baicalin only targets the initial injury—oxidative stress—but not ER stress. Thus, we used tunicamycin-treated HK-2 cells to study the direct effect of Baicalin on ER stress. Tunicamycin is a specific ER stress inducer, due to its specific inhibition of N-linked glycosylation located in the ER [31]. We found that Baicalin treatment significantly reduced tunicamycin-induced ER stress (Figure 5), which suggested a direct inhibition of Baicalin against ER stress. Furthermore, we used inhibitors and inducers of ER stress or Nrf2 expression to figure out their influences on Baicalin-mediated protection (Figure 7). Baicalin pretreatment in combination with Tm or brusatol did not have a protective role against H_2_O_2_-induced cytotoxicity. This result suggested that inhibition of ER stress and activation of Nrf2 are essential for Baicalin-mediated protection. Besides, the combination of Baicalin and TUDCA/*t*-BHQ did not have better protective effects than Baicalin alone, indicating that Baicalin pretreatment already inhibited ER stress and induced Nrf2 expression. These findings confirmed the key role of ER stress and Nrf2 in Baicalin-mediated protection.

The targets of Baicalin was still unclear. One research work indicated that Baicalin activates AMPK through the Ca^2+^/CaMKKβ-dependent pathway in HeLa and A549 cells [32]. AMPK plays a key role in cell physiology and also affects cell response to oxidative stress. However, no research has found the direct relationship between the renal protective effects of Baicalin and the activation of the Ca^2+^/CaMKKβ-AMPK pathway, till now. PPARγ has also been suggested to be one target of Baicalin [33]. In aged rat kidney, Baicalin was found to activate PPARγ and to suppress downstream NF-κB-mediated inflammation. Other studies suggested some potential targets, as well, including the proteasome [34], macrophages [35] and notch signaling [36]. More work is need to clarify the targets of Baicalin.

## 4. Experimental Section

### 4.1. Materials and Reagents

The human renal proximal tubular cell line HK-2 (a kind gift from Honghong Chen, Institute of Radiation Medicine, Fudan University) was adopted in this study. Dulbecco Modified Eagle Medium (DMEM)/F12 and fetal bovine serum (FBS) were purchased from Gibco Technologies (Logan, UT, USA). Baicalin was purchased from Sigma-Aldrich (St. Louis, MO, USA) and diluted in 10% dimethyl sulfoxide (DMSO) (Sigma, St. Louis, MO, USA) before use. Tunicamycin was purchased from Abcam (Cambridge, MA, USA). Brusatol was purchased from JONLN (Shanghai, China). TUDCA was purchased from TCI (Shanghai, China). *tert*-Butylhydroquinone (*t*-BHQ) was purchased from Sigma-Aldrich (St. Louis, MO, USA). The Cell Counting Kit 8 (CCK-8), GSH/GSSG assay kit, Reactive Oxygen Species (ROS) assay kit, Nuclear and Cytoplasmic Protein Extraction kit and Hoechst Staining kit were purchased from Beyotime (Shanghai, China). The TUNEL assay kit was purchased from Roche Applied Science (Indianapolis, IN, USA). The caspase-3 colorimetric assay kit was purchased from KeyGEN (Nanjing, China). TRIzol reagent, superscript II reverse transcriptase and random primer oligonucleotides were purchased from Invitrogen (Carlsbad, CA, USA). The Absolute QPCR SYBR Green premix was purchased from Takara (Otsu, Shiga, Japan). The sequences of primers used in this study are presented in Table 1. The antibodies against BiP, CHOP, cleaved casase-3 and β-actin were purchased from Cell Signaling (Beverly, MA, USA), and the antibodies against Nrf2 and Lamin B were purchased from Santa Cruz (Santa Cruz, CA, USA).

**Table 1:** The sequence of gene-specific primers.

Gene	The Sequence of Gene-Specific Primer
*BiP*	AAAGAAGACGGGCAAAGATGTTGCTTGATGCTGAGAAGACAG
*CHOP*	ACCACTCTTGACCCTGCTTCTCTCTGGGAGGTGCTTGTGAC
*β-actin*	GTTGTCGACGACGAGCG GCACAGAGCCTCGCCTT

### 4.2. Cell Culture and H_2_O_2_ Treatment

HK-2 cells were cultured in DMEM/F12 medium, supplemented with 10% FBS and kept at 37 °C in 5% CO_2_ atmosphere (Heraeus, Hanau, German). Confluent monolayers (80%) of HK-2 cells grown in 6-well plates were treated with or without 500 μmol/L H_2_O_2_ (Lingfeng, Shanghai, China) diluted in serum-free media. Four hours after the exposure, the cells were washed three times with serum-free DMEM/F12 medium and harvested for further analysis. In the treatment groups, Baicalin was added 1 h before H_2_O_2_ stimulation.

### 4.3. Cell Viability Analysis

All groups of cultured HK-2 cells were washed twice with PBS (GNW, Hangzhou, China). The original medium was then replaced by full cultured medium containing 10% WST-8. They were incubated at 37 °C for 1 h to form water dissoluble formazan. Then, the absorbance was measured at 450 nm using a microplate reader (MDC, Hayward, CA, USA). Full cultured medium containing 10% WST-8 was used as a negative control.

### 4.4. Caspase-3 Activity Assay

Relative caspase-3 activity in HK-2 cells was detected with a caspase-3 colorimetric assay kit according to the manufacturer’s instructions. OD was measured at 405 nm with a microplate reader.

### 4.5. Hoechst Staining

HK2 cells were fixed and stained with Hoechst, then examined under a phase contrast microscope equipped with appropriate fluorescence filters (Olympus, Tokyo, Japan). Apoptotic cells are stained brighter, because of their condensed chromatin.

### 4.6. Transferase-Mediated dUTP-Biotin Nick End Labeling (TUNEL) Staining

The terminal deoxynucleotidyl TUNEL assay kit was used to detect apoptotic cells according to the manufacturer’s instructions. Positive-control sections were from a hepatocarcinoma. Apoptotic cells were stained with green fluorescence.

### 4.7. Measurement of Oxidative Stress

The ROS level directly reflects the oxidative stress in cells. GSH and GSSG are important intracellular thiols. Alterations in the GSH/GSSG ratio are used to assess the exposure of cells to oxidative stress. The ROS level of cultured cells was measured using DCFH-DA, and the GSH/GSSG ratio was determined using the GSH/GSSG assay kit (Beyotime, Nantong, China), according to the protocols of the assay kits.

### 4.8. Western Blot Analysis

HK-2 cells were washed twice with PBS and then harvested. Extranuclear and intranuclear proteins were extracted separately according to the protocol of the protein extraction kit. Western blot was performed as previously described [15]. Cleaved caspase-3, BiP, CHOP and Nrf2 were detected with specific antibodies, and their expressions were analyzed after being normalized to references (β-actin in cytoplasm or Lamin B inside nucleus).

### 4.9. Quantitative Real-Time Polymerase Chain Reaction

Total RNA was extracted from cultured cells with TRIzol reagent (Invitrogen, Shanghai, China) according to the manufacturer’s instructions. Total RNA (3–5 μg) was transcribed into cDNA. Our gene-specific primers for human BiP, CHOP and β-actin are listed in Table 1. A real-time quantitative polymerase chain reaction was performed in a Eppendorf Mastercycler ep realplex system (Eppendorf, Hamburg, German) in combination with the Absolute QPCR SYBR Green premix. With a hot start (15 min at 95 °C), the amplification protocol consists of denaturation for 1 s at 95 °C, annealing for 5 s at 60 °C and extension for 10 s at 72 °C for 45 cycles. Expression levels were normalized relative to those of β-actin in the same samples using the 2^−ΔΔ*C*t^ method.

### 4.10. Immunocytochemistry

Cells grown on cover slips were fixed in 4% paraformaldehyde for 10 min, cold preserved overnight at 4 °C and then incubated in PBS for another 30 min at room temperature. Cells were then incubated in 5% BSA in PBS for 1 h at room temperature. The cells were incubated with the primary antibody of Nrf2 for 60 min. After three serial rinses with PBS, the primary antibody was detected with secondary antibodies conjugated to fluorescein isothiocyanate. Finally, the nuclei were counterstained with 1 mg/mL 4,6-diamidino-2-phenylindole. Negative controls were obtained by following the same protocol while omitting the primary antibodies. Samples were examined under a phase contrast microscope equipped with appropriate fluorescence filters.

### 4.11. Statistical Analysis

Data are presented as the mean ± SEM. The results in two groups were compared using two-tailed independent *t*-tests, and the results among three or more groups were compared by one-way analysis of variance. All statistical analysis were performed using SPSS 13.0, with *p* < 0.05 considered statistically significant.

## 5. Conclusions

Our experiments proved that Baicalin pretreatment alleviates H_2_O_2_-induced cytotoxicity in HK-2 cells. The underlying mechanism is related to the inhibition of ER stress and the activation of downstream Nrf2 signaling.
